# Exploring the Chemical Profile, In Vitro Antioxidant and Anti-Inflammatory Activities of *Santolina rosmarinifolia* Extracts

**DOI:** 10.3390/molecules29071515

**Published:** 2024-03-28

**Authors:** Janos Schmidt, Kata Juhasz, Agnes Bona

**Affiliations:** Department of Biochemistry and Medical Chemistry, Medical School, University of Pécs, 7624 Pécs, Hungary; janos.schmidt@aok.pte.hu (J.S.); kata.juhasz@aok.pte.hu (K.J.)

**Keywords:** *Santolina rosmarinifolia*, LC-MS, ABTS, DPPH, Folin–Ciocalteu assay, TPC, NO production, Griess assay

## Abstract

In this study, the phytochemical composition, in vitro antioxidant, and anti-inflammatory effects of the aqueous and 60% ethanolic (EtOH) extracts of *Santolina rosmarinifolia* leaf, flower, and root were examined. The antioxidant activity of *S. rosmarinifolia* extracts was determined by 2,2’-azino-bis(3-ethylbenzothiazoline-6-sulfonic acid) (ABTS) and 2,2-diphenyl-1-picrylhydrazyl (DPPH) radical scavenging assays. The total phenolic content (TPC) of the extracts was measured by the Folin–Ciocalteu assay. The anti-inflammatory effect of the extracts was monitored by the Griess assay. The chemical composition of *S. rosmarinifolia* extracts was analysed using the LC-MS technique. According to our findings, 60% EtOH leaf extracts showed the highest Trolox equivalent antioxidant capacity (TEAC) values in both ABTS (8.39 ± 0.43 µM) and DPPH (6.71 ± 0.03 µM) antioxidant activity assays. The TPC values of the samples were in good correspondence with the antioxidant activity measurements and showed the highest gallic acid equivalent value (130.17 ± 0.01 µg/mL) in 60% EtOH leaf extracts. In addition, the 60% EtOH extracts of the leaves were revealed to possess the highest anti-inflammatory effect. The LC-MS analysis of *S. rosmarinifolia* extracts proved the presence of ascorbic acid, catalpol, chrysin, epigallocatechin, geraniol, isoquercitrin, and theanine, among others, for the first time. However, additional studies are needed to investigate the direct relationship between the chemical composition and physiological effects of the herb. The 60% EtOH extracts of *S. rosmarinifolia* leaves are potential new sources of natural antioxidants and anti-inflammatory molecules in the production of novel nutraceutical products.

## 1. Introduction

Species related to the *Santolina* genus have been used as traditional medicines in the Mediterranean area of Europe, particularly in Italy and Spain. These herbs support digestion and are characterised by anti-inflammatory, spasmolytic, antibacterial, and analgesic activities [1]. *S. rosmarinifolia*, also known as green lavender cotton, is an evergreen with feathery green leaves and button-shaped, numerous yellow flowers. Its pleasant, chamomile-like fragrance is attributed to the abundant volatile oil content of the plant.

Most of the previous studies focused predominantly on analysing volatile components in the essential oil of *Santolina* species using gas chromatography–mass spectrometry (GC-MS) [1]. For instance, Ahmed et al. [2] identified artemisia ketone, *Z*-thujone, (2*Z*,6*E*)-farnesol, and limonene as the primary volatile constituents in the aerial flowering parts of *S. chamaecyparissus L.* In the study by Ioannou et al. [3], the essential oil of *S. rosmarinifolia* flower heads contained β-eudesmol, 1,8-cineole, camphor, borneol, *ar*-curcumene, terpinen-4-ol, and spathulenol in significant amounts. Conversely, the dominant volatile constituents in *S. rosmarinifolia* leaves were *ar*-curcumene, β-phellandrene, spathulenol, β-pinene, γ-muurolene, myrcene, and camphor. Palá-Paúl et al. [4] used GC-MS to monitor the monthly changes in the phytochemical composition of *S. rosmarinifolia* aerial parts and discovered that the capillene concentration was dependent on precipitation, while levels of β-phellandrene, limonene, and 1,8-cineole were temperature-dependent.

While most studies have focused on GC-MS, a few have explored the chemical composition of *Santolina* plant extracts using high-performance liquid chromatography (HPLC). Notably, the methanolic extracts of *S. corsica* were found to contain kaempferol-3-*O*-glucoside, chlorogenic acid, and rosmarinic acid [5], while the ethanolic extract of *S. semidentata* was characterised by β-pinene, E-pinocarveol, and pinocarvone as the main constituents [6].

Moreover, liquid chromatography–mass spectrometry (LC-MS) has successfully identified chlorogenic acid and cynarin in the aqueous extracts of *S. impressa* leaves and flowers, respectively [7]. Conversely, Sánchez-Vioque et al. [8] identified quercetin-3-*O*-glucoside, apigenine, and hydroxycinnamoylquinic acid as the main components in the solid residue of *S. rosmarinifolia* after steam distillation. Boudoukha et al. [9] found that the methanolic extract of *S. chamaecyparissus* contained mostly caffeic acid, *p*-coumaric acid, luteolin-7-*O*-glucoside, rutin, luteolin, and quercetin. Aourach et al. [10] reported that the aqueous extracts of *S. chamaecyparissus* were rich in cynarin and chlorogenic acid, while Azevedo et al. [11] identified cynarin and myricetin-*O*-glucuronide as the major phenolic compounds of the same species. Additionally, Messaoudi et al. [12] detected chlorogenic acid and apigenin-7-glycoside as the most abundant components in both the aqueous and ethanolic extracts of *S. chamaecyparissus.*

It is well known that the increased production of reactive oxygen species (ROS) leads to oxidative stress, which can play a crucial role in the formation of chronic diseases (such as cancer, coronary heart diseases, and neurological disorders) [13]. Antioxidant capacity assays are frequently used to measure the free radical scavenging activity of medicinal plants. The in vitro antioxidant activity of various *Santolina* species has been studied by 2,2’-azino-bis(3-ethylbenzothiazoline-6-sulfonic acid) (ABTS) [5], 2,2-diphenyl-1-picrylhydrazyl (DPPH) [5,7,8,14], oxygen radical absorbance capacity (ORAC) [6], and ferric reducing activity power (FRAP) [5,8,14] methods. The herb extracts analysed in these studies showed promising but concentration- and solvent-dependent radical scavenging activity.

The concentration of phenolic compounds with potential antioxidant activities can be measured by the Folin–Ciocalteu assay and expressed in gallic acid equivalent (GAE) [15,16]. The total phenolic content (TPC) of plants related to *Santolina* species has been studied, and a relatively high antioxidant concentration was observed [14,17,18]. However, the GAE values of the samples greatly varied according to species [14] and the type of extraction procedure [18].

*Santolina* species are also known for their anti-inflammatory effect. Lipopolysaccharide (LPS) found in bacterial cell walls induces fever, inflammation, and septic shock by stimulating the synthesis of proinflammatory cytokines. The LPS-induced production of the inflammatory mediator nitric oxide (NO) in RAW 264.7 macrophages is one of the most frequently used methods to monitor the anti-inflammatory activity of herb extracts. LPS activates the nuclear factor kappa B (NFkB) by triggering Toll-like receptors (TLRs) of the macrophages, in turn elevating the expression of inducible nitric oxide synthase (iNOS). iNOS is a proinflammatory enzyme responsible for NO overproduction. Chronic inflammatory diseases such as arthritis, bowel diseases, atherosclerosis, and cancer are associated with similar proinflammatory enzyme pathways [19]. Active phytochemicals present in the extracts of other *Santolina* species suppressed NO production in LPS-treated murine macrophage cells [20,21,22], through the downregulation of the NF-kB pathway, highlighting their potential therapeutic use.

The aim of this study was to evaluate the detailed chemical composition, antioxidant, and anti-inflammatory activity of *S. rosmarinifolia* leaf, flower, and root extracts to verify the traditional use of the herb and to support its future use as a therapeutic agent. Previous studies did not analyse the individual free radical scavenging and anti-inflammatory potential of different plant parts of the *Santolina* genus; furthermore, there is a lack of studies dealing with *S. rosmarinifolia* species. We also analysed whether 60% ethanolic (EtOH) or aqueous extracts of the herb exert higher free radical scavenging and anti-inflammatory activities. Our further goal was to provide more information about the phytochemical profile of the different plant parts and identify chemical compounds that were not yet described in *S. rosmarinifolia*.

## 2. Results and Discussion

### 2.1. Antioxidant Activity Assays

#### 2.1.1. Trolox Equivalent Antioxidant Capacity (TEAC)

**Figure 1:**
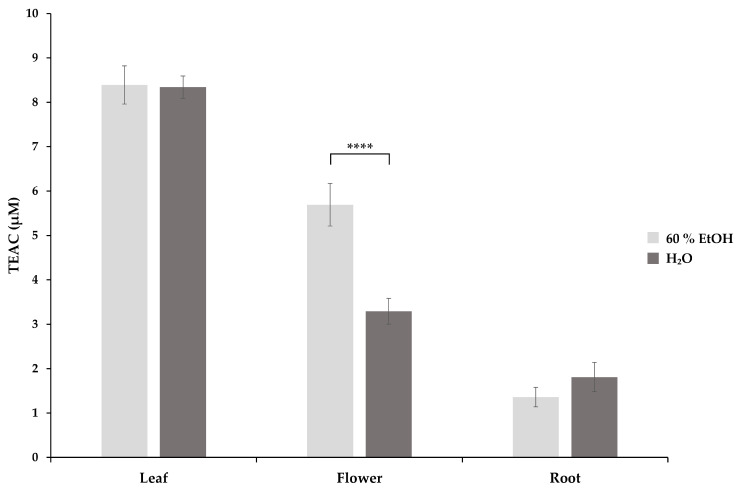
The ABTS radical scavenging activity of *S. rosmarinifolia* 60% EtOH and aqueous extracts expressed as Trolox equivalent antioxidant capacity (TEAC) in micromolar concentration (µM). The error bars show the standard deviation calculated from a triplicate of samples. Significance levels were determined using two-way ANOVA with Tukey’s test and are indicated as **** = *p* < 0.0001.

The antioxidant activity of the extracts was assessed by an in vitro ABTS test, where the antioxidants present in the samples act as reducing agents for ABTS^•+^, inducing a decolourisation reaction [16]. The Trolox equivalent antioxidant capacity (TEAC) of aqueous and 60% EtOH extracts of *S. rosmarinifolia* leaves, flowers, and roots were determined (Figure 1). Two-way ANOVA with Tukey’s test showed a significant difference in the mean TEAC of the different plant parts (*p* < 0.01). Leaf extracts with the highest TEAC values both in aqueous (8.34 ± 0.25 µM) and 60% EtOH (8.39 ± 0.43 µM) extracts were observed to have similar means. Flower extracts exerted a significantly lower antioxidant activity in aqueous (5.69 ± 0.48 µM) as well as in 60% EtOH (3.29 ± 0.29 µM) extracts. Aqueous (1.36 ± 0.22 µM) and 60% EtOH (1.81 ± 0.33 µM) root extracts showed the lowest antioxidant capacity. Comparing the TEAC values of the corresponding aqueous and 60% EtOH extracts, only the 60% EtOH flower extract showed a significantly higher TEAC value than its aqueous extract.

Others have also found a significant and solvent-dependent antioxidant capacity of *Santolina* species. The methanolic extract of *S. corsica* [5] and *S. africana* [14,17] aerial parts showed a significantly high ABTS antioxidant capacity. The methanolic [9] and ethyl acetate extracts [23], as well as butanol [18,23] fractions of *S. chamaecyparissus*, proved to have excellent ABTS-reducing power.

To the best of our knowledge, no other study has explored the ABTS radical scavenging activity of ethanolic or aqueous extracts from the leaves, flowers, and roots of plants belonging to the *Santolina* genus. The results indicate that both solvents are effective for extracting bioactive molecules from the most promising leaf samples. The introduction of lyophilisation for various plant parts before the sample preparation procedure, along with measuring the ABTS antioxidant capacity of *S. rosmarinifolia* as a potential source of natural antioxidant compounds, adds novelty to our current study.

#### 2.1.2. DPPH Antioxidant Activity Assay

**Figure 2:**
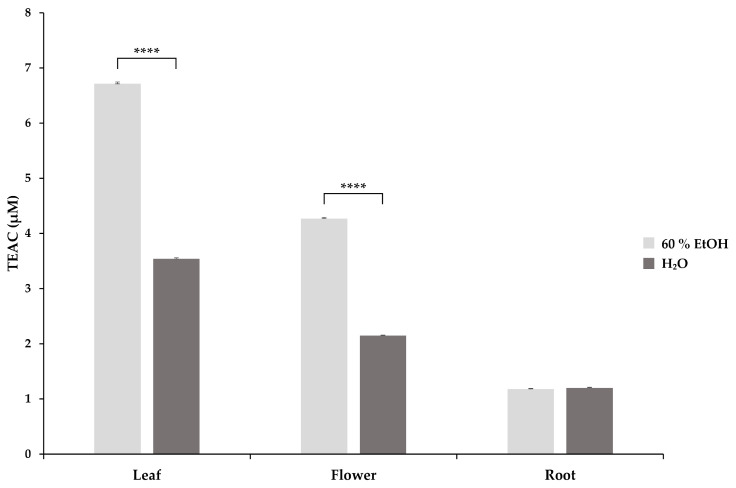
The DPPH radical scavenging activity of *S. rosmarinifolia* 60% EtOH and aqueous extracts expressed as Trolox equivalent antioxidant capacity (TEAC) in micromolar concentration (µM). The error bars show the standard deviation calculated from a triplicate of samples. Significance levels were determined using two-way ANOVA with Tukey’s test and are indicated as **** = *p* < 0.0001.

DPPH, a stable free radical, is reduced by antioxidant molecules, which results in a colour change from purple to yellow and is monitored at 517 nm [2]. The TEAC values of *S. rosmarinifolia* aqueous and 60% EtOH extracts were calculated (Figure 2). Two-way ANOVA with Tukey’s test showed a significant difference in the mean TEAC of the different plant parts (*p* < 0.0001). Similar to ABTS measurements, leaf extracts showed the highest TEAC values both in aqueous (3.54 ± 0.02 µM) and 60% EtOH (6.71 ± 0.03 µM) extracts, while flower extracts exerted lower antioxidant activity in aqueous (2.15 ± 0.01 µM) as well as in 60% EtOH (4.27 ± 0.02 µM) solutions. Aqueous (1.20 ± 0.01 µM) and 60% EtOH (1.18 ± 0.01 µM) root extracts showed the lowest free radical scavenging activity. Comparing the TEAC values of the corresponding aqueous and 60% EtOH extracts, we found that the 60% EtOH leaf and flower extracts had significantly higher antioxidant activity.

The ethyl acetate [23] and chloroformic extracts [18] of *S. chamaecyparissus* aerial parts exerted high, concentration-dependent DPPH scavenging activity, as well as the methanolic extracts of *S. africana* [17]. Essential oils of *S. chamaecyparissus* [2,14] and *S. angustifolia* [14] extracted by hydrodistillation from whole plants exerted a significant antioxidant potential as well.

The individual DPPH antioxidant capacity in leaf, flower, and root samples of *Santolina* species has not been studied yet. However, exploring this aspect could provide valuable information about which part exhibits the highest antioxidant activity, making it a promising ingredient for novel nutraceutical products. According to our results, an ethanolic extraction procedure using only the leaves of the plant is worthy of consideration. We utilised 60% EtOH as a solvent and employed freeze-drying on *S. rosmarinifolia* samples for the first time, demonstrating a more efficient extraction of biomolecules with potential antioxidant effects from the leaves compared to aqueous extracts, despite the higher temperature used for the extraction of the aqueous samples.

### 2.2. Determination of Total Phenolic Content

**Figure 3:**
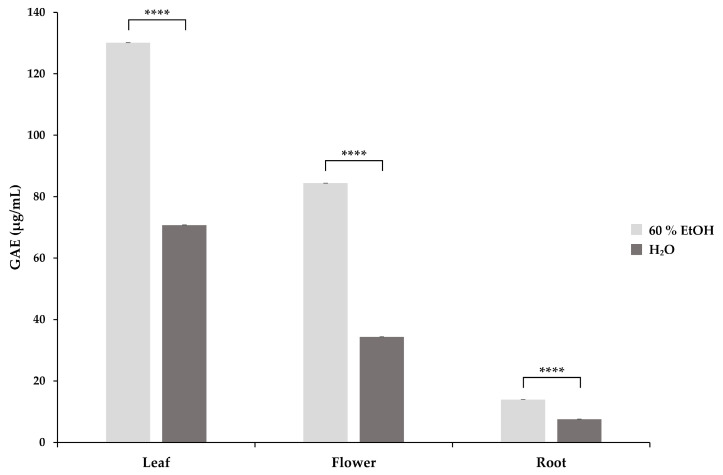
The total phenolic content (TPC) of *S. rosmarinifolia* 60% EtOH and aqueous extracts expressed as gallic acid equivalent (GAE) in µg/mL. The error bars show the standard deviation calculated from a triplicate of samples. Significance levels were determined using two-way ANOVA with Tukey’s test and are indicated as **** = *p* < 0.0001.

The GAE values of *S. rosmarinifolia* aqueous and 60% EtOH extracts are presented in Figure 3. Two-way ANOVA with Tukey’s test showed a significant difference in the mean TEAC of the different plant parts (*p* < 0.0001). Based on our measurements, leaf extracts showed the highest TPC expressed in GAE (µg/mL) both in aqueous (70.74 ± 0.02 µg/mL) and 60% EtOH (130.17 ± 0.01 µg/mL) extracts, while flower extracts showed lower values in aqueous (34.35 ± 0.01 µg/mL) as well as in 60% EtOH (84.42 ± 0.02 µg/mL) extracts. Aqueous (7.49 ± 0.05 µg/mL) and 60% EtOH (13.94 ± 0.01 µg/mL) root extracts showed the lowest phenolic content. Comparing the GAE values of the corresponding aqueous and 60% EtOH extracts, we found that the 60% EtOH extracts had a significantly higher antioxidant content in each case.

The TPC of *S. chamaecyparissus* aerial parts was significant in ethyl acetate [23] and butanol [18] extracts. The methanolic extracts of *S. africana* also showed high TPC [17]. The essential oils of *S. chamaecyparissus* [2,14] and *S. angustifolia* [14] are rich sources of polyphenols as well.

In good correspondence with the performed antioxidant capacity measurements (ABTS, DPPH), our findings related to the total phenolic content of the herb extracts suggest that the 60% EtOH extraction of the freeze-dried leaf samples instead of the extraction of the whole plant could increase the concentration of phenolic compounds in the extract.

### 2.3. Inhibition of Nitric Oxide (NO) Production

**Figure 4:**
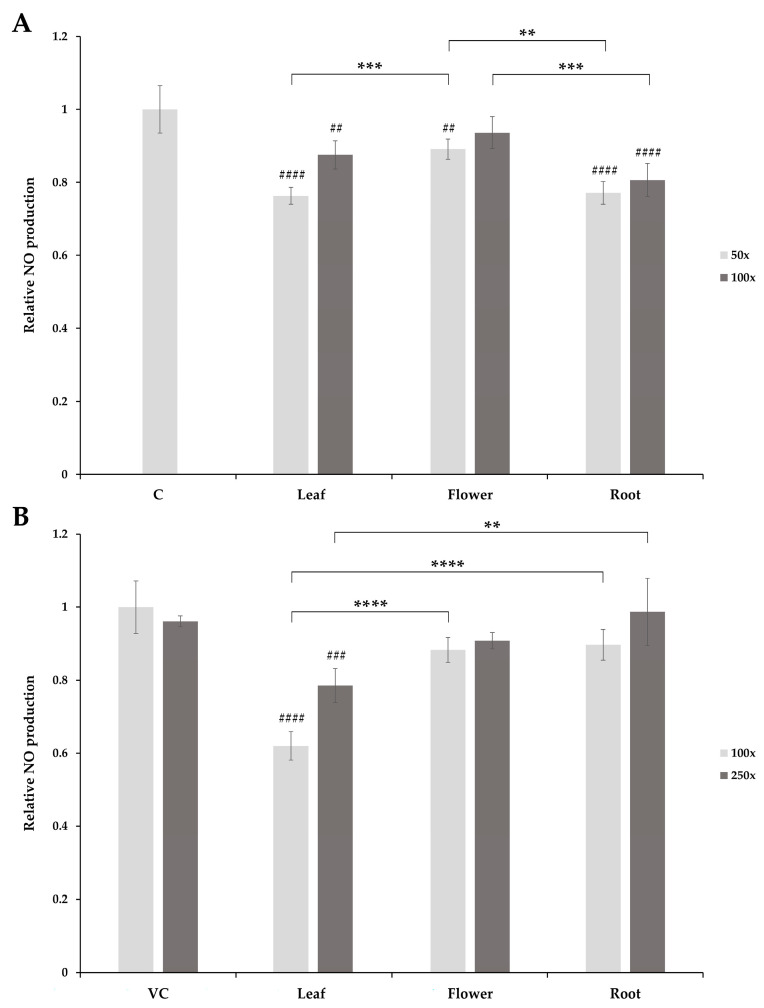
Anti-inflammatory activity of aqueous and 60% EtOH *S. rosmarinifolia* extracts: (**A**) Effect of *S. rosmarinifolia* aqueous extracts on NO production in LPS-treated RAW 264.7 cells. Murine macrophage cells were pretreated with the indicated dilutions of leaf, flower, or root stock extracts of *S. rosmarinifolia* for 6 h, and then cells were challenged by 1 µg/mL LPS for 24 h. NO production was measured using the Griess reagent. (**B**) Effect of *S. rosmarinifolia* 60% EtOH extracts on NO production in LPS-treated RAW 264.7 cells. Murine macrophage cells were pretreated with the indicated dilutions of leaf, flower, or root stock extracts of *S. rosmarinifolia* for 6 h, and then cells were challenged by 1 µg/mL LPS for 24 h. NO production was measured using the Griess reagent. The error bars show the standard deviation calculated from five parallel samples. Significance levels were determined using two-way ANOVA with Tukey’s test and are indicated as ** = *p* < 0.01, *** = *p* < 0.001, **** = *p* < 0.0001, ^##^ = *p* < 0.01, ^###^ = *p* < 0.001, ^####^ = *p* < 0.0001.

To assess the influence of the extracts on LPS-induced NO production in RAW 264.7 macrophages, cells were pretreated with herb extracts of different concentrations for 6 h, and then cells were stimulated by 1 µg/mL LPS for 24 h. The NO production of the treated cells was measured by the Griess assay. The pretreatment of the cells with 50- and 100-fold dilutions of leaf and root aqueous extracts showed a significant decrease in NO production upon LPS challenge compared with the control cells (vehicle control (VC), control (C)). The 50-fold dilution of the aqueous flower extracts also showed significant inhibition, while no significant inhibition could be observed with the 100-fold dilution (Figure 4A). The 60% EtOH extracts of leaf samples significantly inhibited NO production in LPS-treated cells, both in 100- and 250-fold dilutions, while no inhibition could be observed with the 60% EtOH extracts of flower and root samples (Figure 4B).

Comparing the NO inhibition of the corresponding 60% EtOH and aqueous extracts in 100-fold dilutions, a significant difference was observed in the case of leaf samples (*p* < 0.0001).

Similar changes in the NO production of LPS-treated RAW 264.7 macrophage cells have been reported upon pretreatment with essential oils or extracts of different *Santolina* species. Alves-Silva et al. [20] measured the anti-inflammatory activity of essential oils extracted from the flowering stems of *S. impressa* by hydrodistillation. Significantly decreased NO levels were found in macrophages pretreated with different concentrations of *S. impressa* essential oil, and the NO inhibition increased in a concentration-dependent manner. According to the findings of the same research group, the essential oil content of *S. rosmarinifolia* arial parts exerted the same effect on murine macrophage cells downregulating LPS-triggered NO production [21]. The methanolic and n-hexane extracts of the aerial parts of *S. pinnata* also showed a dose-dependent anti-inflammatory effect on the same cell line [22]. In contrast, the methanolic extract of *S. corsica* aerial parts was not able to inhibit NO production; however, the n-hexane extract of the same herb concentration-dependently suppressed the NO levels in LPS-stimulated RAW 264.7 cells [5].

The above studies mostly dealt with the inhibitory effect of the essential oils yielded from *Santolina* species. The inhibitory effect of the ethanolic or aqueous extracts of the individual parts of *S. rosmarinifolia* was unknown. Based on our findings, *S. rosmarinifolia* leaves extracted with 60% EtOH exerted significant anti-inflammatory activities even in lower concentrations, which was in a good correlation with the antioxidant capacity and phenolic content of the samples, further supporting the use of these extracts as a possible bioactive ingredient in dietary supplements.

### 2.4. Chemical Composition of S. rosmarinifolia Extracts

**Figure 5:**
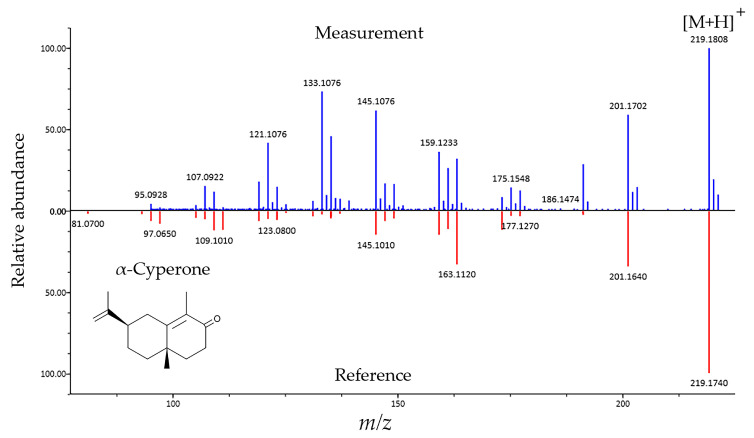
Identification of α-cyperone in *S. rosmarinifolia* using MS-DIAL ESI(+)-MS/MS database from authentic standards. The MS^2^ fragment spectrum of the detected component (measurement) was matched (match score: 1.5827) with the MS^2^ fragment spectrum of the molecule from the database (reference).

The chemical composition of the plant was determined using LC-MS. The MS^2^ fragmentation profiles obtained from the shotgun metabolomic procedure were processed with the assistance of the MS-DIAL program tool. Figure 5 shows the match of the fragmentation profile of one of the identified components (α-cyperone) compared to the MS^2^ profile present in the MS-DIAL database.

**Figure 6:**
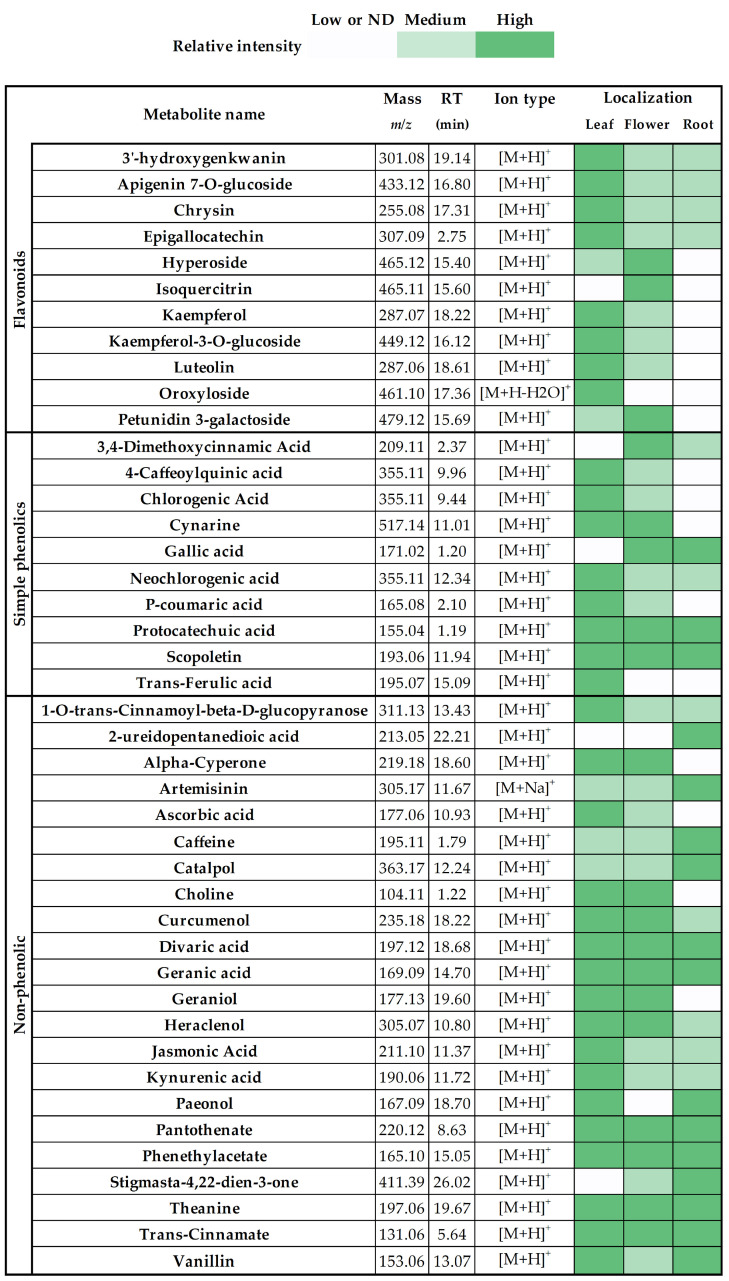
The identified components and their relative abundance in *S. rosmarinifolia* leaf, flower, and root extracts. The colours represent the ion intensity of individual components; low or ND (white) indicate molecules with relative intensity values < 1 × 10^3^, medium (light green) includes molecules within the relative intensity range of 1 × 10^3^ and 1 × 10^4^, and those above 1 × 10^4^ relative intensity values are marked as high (dark green).

The identified components in *S. rosmarinifolia* leaf, flower, and root extracts and their relative abundance are shown in Figure 6. Different colours illustrate the relative distribution of particular molecules in different plant parts. Based on their ion intensity values, they were labelled as low or not detected, medium, or high, referring to their distribution in the plant.

Previous studies also identified apigenin 7-*O*-glucoside [24], chlorogenic acid [25], cynarine [7], gallic acid [5], kaempferol [22], kaempferol 3-*O*-glucoside [5], luteolin [22], neochlorogenic acid [5], p-coumaric acid [9], protocatechuic acid [5], scopoletin [26], stigmasta-4,22-dien-3-one [5], trans-ferulic acid [22] and vanillin [27] in different *Santolina* species. To the best of our knowledge, ascorbic acid, catalpol, chrysin epigallocatechin, geraniol, isoquercitrin, and theanine, among other compounds (Figure 6), were identified for the first time in the *Santolina* genus. The newly identified molecules may also contribute to the beneficial effects of the herb; however, further studies are required to measure the individual anti-inflammatory and antioxidant effects of the identified compounds isolated from *S. rosmarinifolia*.

## 3. Materials and Methods

### 3.1. Chemicals and Reagents

Standards of ABTS^•+^, Trolox, DPPH, Folin–Ciocalteu reagent, and gallic acid, as well as sodium carbonate, ammonium formate, and potassium persulfate, were purchased from Merck Life Science Kft. (Budapest, Hungary). For the preparation of the solutions and eluents, hypergrade LC-MS ethanol, methanol, water, and formic acid (Merck Life Science Kft., Budapest, Hungary) were used. RAW 264.7 cell lines (TIB-71) were purchased from ATCC (Massas, VA, USA). Dulbecco’s modified Eagle’s medium (DMEM) was obtained from Biosera (Nuaille, France), and 10% ultralow endotoxin foetal bovine serum (FBS) was obtained from GIBCO (New York, NY, USA). The Griess reagent was purchased from Thermo Fisher Scientific (Darmstadt, Germany).

### 3.2. Plant Materials and Extraction Procedure

*S. rosmarinifolia* samples were collected during the flowering stage in June 2023 in Pécs, Hungary (46.080873° N, 18.245665° E). The leaves, flowers, and roots of the plants were separated manually and freeze-dried. The taxonomic identity of the plant was confirmed by Dr Tamás Wirth, and a voucher specimen was preserved at the Herbarium of Botanical Garden (JPU), Faculty of Sciences, University of Pécs, Pécs, Hungary. Lyophilised plant parts (50 mg) were extracted with 1 mL of water or 1 mL of 60% EtOH. The samples were homogenised using a Sonics Vibra-Cell ultrasonic liquid processor and then shaken at 60 °C (60% EtOH extracts) and 80 °C (aqueous extracts) for 30 min (Eppendorf Thermomixer Comfort, Hamburg, Germany). Afterwards, the extracts were centrifuged at 13,000× *g* for 15 min (Eppendorf Centrifuge 5430 R, Hamburg, Germany) at room temperature and finally filtered through Acrodisc syringe filters (0.2 μm) (Pall Life Sciences, New York, NY, USA). For the Griess assay, the solvent content of the samples (200 µL) was removed with a SpeedVac evaporator (Eppendorf Concentrator Plus, Hamburg, Germany). The samples were stored at −80 °C until further measurements.

### 3.3. Antioxidant Activity Assays

#### 3.3.1. ABTS Antioxidant Activity Assay

The antioxidant activity of the samples was determined using the ABTS method according to Re et al. [16]. The prepared ABTS^•+^ stock solution was diluted with ethanol to set the absorbance to 0.70 (±0.05) at 734 nm. The diluted ABTS^•+^ solution was used for the dilution of the herb extracts to achieve a 20–90% inhibition of the blank absorbance. Two µL of leaf, flower, and root stock solutions (50 mg dry weight/mL) were diluted to 1000-fold with 2 mL of ABTS^•+^ solution. The Trolox standard (1 mg) was dissolved in 1 mL of ethanol. The final concentrations of Trolox standards in the diluted ABTS^•+^ solution were 1, 2, 4, 6, 8, and 10 µM. For the preparation of the solutions, LC-MS grade quality ethanol and water were applied. Absorption measurements were taken at room temperature, 1 min after mixing herb extracts/Trolox standards and ABTS^•+^ solution. The spectrophotometric measurements were performed with a Jasco spectrophotometer V-550 UV/Vis. All determinations were carried out in triplicate. The percentage inhibition values of Trolox solutions at 734 nm were calculated as follows:

% inhibition = ((A_0_ − A_antioxidant_)/A_0_) × 100 (1)

where A_0_ is the absorbance of the ABTS^•+^ solution, and A_antioxidant_ is the absorbance measured after the addition of the antioxidant. The Trolox equivalent antioxidant capacity (TEAC) values of the extracts were calculated.

#### 3.3.2. DPPH Antioxidant Activity Assay

The DPPH radical scavenging activity of the different plant extracts was measured using the method described by Ahmed et al. [2], with some modifications. The DPPH solution (150 µM) was prepared in ethanol. Then, 2 µL *S. rosmarinifolia* extracts (50 mg dry weight/mL) were added to the DPPH solution (2 mL) and vortexed thoroughly. After 30 min at room temperature, the absorbance was measured at 517 nm. The Trolox standard (1 mg) was dissolved in 1 mL of ethanol. The final concentrations of Trolox in DPPH were 1, 2, 4, 6, 8, and 10 µM. All determinations were carried out in triplicate. The percentage inhibition values of Trolox solutions at 517 nm were calculated as follows:

% inhibition = ((A_0_ − A_antioxidant_)/A_0_) × 100 (2)

where A_0_ is the absorbance of the DPPH solution and A_antioxidant_ is the absorbance measured after the addition of the antioxidant. The Trolox equivalent antioxidant capacity (TEAC) values of the extracts were calculated.

### 3.4. Determination of Total Phenolic Content

The TPC of the plant extracts was determined using the Folin–Ciocalteu method according to Li et al. [28], with some modifications. Folin–Ciocalteu reagent (5 mL) was diluted with distilled water to 10-fold. Afterwards, the prepared reagent (1980 µL) was mixed with 20 µL of the samples (50 mg dry weight/mL). A sodium carbonate solution (1 mL) of 4 *w*/*v*% was then added to the mixture. After 120 min at room temperature, the absorbance of the samples was measured at 765 nm. Gallic acid in 5, 25, 50, 100, 125, and 150 µg/mL concentrations was used as a standard. The results were expressed in GAE (µg/mL). All determinations were carried out in triplicate.

### 3.5. Cell Culture

The murine leukemic monocyte–macrophage cell line RAW 264.7 was cultured in high-glucose DMEM supplemented with 10% ultralow endotoxin FBS at 37 °C with 5% CO_2_ in a humidified incubator. The evaporation residues obtained from the 60% EtOH and aqueous stock solutions (50 mg dry weight/mL) of the extracts were dissolved in a 200 µL serum-free medium containing 50% EtOH and in a 200 µL serum-free medium, respectively.

### 3.6. Inhibition of Nitric Oxide (NO) Production

The NO production of RAW 264.7 cells was measured by the Griess assay, according to Herraiz et al. [29]. Briefly, RAW 264.7 cells (10^5^ cells/well) were plated into 96-well plates. Cells were pretreated with *S. rosmarinifolia* extracts of different concentrations (for aqueous solutions, 50- and 100-fold dilutions were used, and for 60% EtOH solutions, 100- and 250-fold dilutions of the stock solutions were used) for 6 h and then stimulated with 1 µg/mL LPS in a complete medium for 24 h. Cell supernatants (50 µL) were mixed with 50 µL of the Griess reagent and incubated for 10 min at room temperature. The absorbance of the samples was measured at 540 nm by using the Promega GlowMax Multi Plate Reader (Promega, Southampton, UK). All measurements were carried out in five parallels.

### 3.7. Statistical Analysis

All antioxidant capacity measurements were carried out in triplicate, and the NO production of the RAW 264.7 cells was analysed in five parallels. Data were expressed as means ± SD. Statistical analysis was performed by R (version 4.3.2) statistical computing software. The measurement data were analysed by two-way ANOVA followed by a post hoc Tukey test. Differences were considered significant at *p* < 0.05.

### 3.8. LC-MS Analysis

Stock solutions of aqueous and 60% EtOH plant extracts (50 mg dry weight/mL) were transferred into 1.5 mL autosampler glass vials. Chromatographic separation was performed using the Thermo Ultimate 3000 UHPLC™ system (Thermo Fisher Scientific, Waltham, MA, USA) with a Kinetex C18 reversed-phase column (2.6 μm, 2.1 mm × 150 mm i.d.) from Phenomenex (Torrance, CA, USA). For the multistep gradient-based separation method, two different mobile phases were used. Solvent A consisted of water/formic acid (99.9/0.1, *v*/*v*), while solvent B was composed of water/formic acid/methanol (9.9/0.1/90, *v*/*v*/*v*). Both eluents contained 10 mM ammonium formate. The injection volume was 5 µL. The gradient program included the following steps: 0.0–1.0 min 0% B; 1.0–10.0 min from 1.0 to 35.0% B; 10.0–18.0 min from 35.0 to 100% B; 18.0–26.0 min 100% B; 26.0–27.0 min from 100 to 0% B; and 27.0–32.0 min 0% B. The flow rate was 250 µL/min. The data-dependent mass spectrometric acquisition was performed using Bruker Maxis 4G UHR-QTOF instrument (Bruker Daltonics, Bremen, Germany). The mass spectrometer was operated in the positive ion mode, and the scanning range was set to 100–2000 *m*/*z*. The flow of nebuliser gas was 10 L/min at a pressure of 2.5 bar, and the temperature was set to 250 °C. The capillary voltage was 4 kV. The 30 most intensive compounds were selected for CID fragmentation. The collected data were converted to Abf format using the Reifycs file converter tool and processed with MS-DIAL (Version 5.1.230222) software. For database identification, MS-DIAL ESI(+)-MS/MS from authentic standards, ReSpect, MassBank-EU, BMDMS-NP, and Fiehn/Vaniya MSP libraries were used (http://prime.psc.riken.jp/compms/msdial/main.html#MSP, accessed on 21 August 2022). Database identification was performed based on Tsugawa et al. [30]. Molecules were considered as identified if their similarity (match) score exceeded the value of 1.2.

## 4. Conclusions

The present study focused on the total phenolic content, free radical scavenging, and anti-inflammatory activities of *S. rosmarinifolia* leaf, flower, and root extracts. Our results suggest that the 60% EtOH extract of the leaf may be of interest for pharmaceutical use, owing to its demonstrated antioxidant and anti-inflammatory effects. Furthermore, this study identified numerous compounds, including ascorbic acid, catalpol, chrysin, epigallocatechin, geraniol, isoquercitrin, and theanine, for the first time from a plant of the *Santolina* genus. These findings may encourage further studies to investigate the antioxidant and anti-inflammatory effects of individual molecules offered by *S. rosmarinifolia*.

## Data Availability

The data presented in this study are available in the article.
